# The impact of the COVID-19 response on the provision of other public health services in the U.S.: A cross sectional study

**DOI:** 10.1371/journal.pone.0255844

**Published:** 2021-10-14

**Authors:** Kristina W. Kintziger, Kahler W. Stone, Meredith A. Jagger, Jennifer A. Horney

**Affiliations:** 1 Department of Public Health, University of Tennessee, Knoxville, Tennessee, United States of America; 2 Department of Health and Human Performance, Middle Tennessee State University, Murfreesboro, Tennessee, United States of America; 3 Non-affiliated Researcher, Austin, Texas, United States of America; 4 Epidemiology Program, University of Delaware, Newark, Delaware, United States of America; University of the Witwatersrand, SOUTH AFRICA

## Abstract

**Introduction:**

Funding and staff formerly dedicated to routine public health tasks (e.g., responding to communicable and non-communicable diseases, investigating foodborne outbreaks, conducting routine surveillance) and services (e.g., environmental health, substance abuse, maternal-child health) may no longer be available in many public health departments due to the COVID-19 response. The objective of this study was to assess the extent to which staffing for essential public health services has been redirected to the COVID-19 response.

**Materials and methods:**

This is a cross-sectional study using a survey distributed through the Qualtrics platform. Individuals (N = 298) working in public health across governmental and academic public health departments in the U.S. during the ongoing COVID-19 pandemic response were surveyed. Survey items measured multiple domains including professional experience (i.e., training, years of experience, content expertise, job functions, hours worked), mental and physical health status (i.e., generalized anxiety, depression, burnout), and career plans (i.e., pre-pandemic vs. current career plans).

**Results:**

The total number of content expertise areas and programmatic functions covered by individual public health workers increased between January and September of 2020, with 26% (73 of 282) of respondents reporting an increase in both. The total number of respondents working in infectious disease and preparedness remained constant, while declines were reported in program evaluation (-36%) and health education (-27%) and increases were reported in disease investigation (+35%).

**Conclusions:**

The provision of many essential public health functions and tasks have been limited or eliminated while the U.S. public health workforce responds to the COVID-19 pandemic. These findings highlight opportunities for funding and professional development of public health systems, both during and after the COVID-19 response, to help ensure the continuity of essential public health services, staffing sustainability, and preparedness for future public health emergencies in the U.S.

## Introduction

COVID-19 has had major global impacts on many professionals, including the frontline patient-facing workforce caring for those infected as well as public health staff managing the implementation of control measures including testing programs, isolation and quarantine, contact tracing, and vaccination programs. As of the end of December 2020, 79 million cases and 1.7 million deaths have been reported worldwide [1]; of these, 21 million cases and over 360,000 deaths occurred in the U.S. alone [2].

Among frontline healthcare workers, the direct impacts of the COVID-19 pandemic response on mental and physical health have been well-documented [3–10]. However, there are other important indirect impacts of COVID-19 on the healthcare workforce. Hospitals across the U.S. have faced staffing shortages, particularly during COVID-19 surges, which have required transferring patients over long distances for care [11]. Workforce shortages have also led to longer shifts for many healthcare workers, particularly nurses, an important component of the healthcare workforce severely short-staffed before the pandemic [12].

The public health workforce [13] includes epidemiologists and other public health practitioners who respond to public health threats through surveillance (e.g., testing), investigation (e.g., contact tracing), and prevention (e.g., vaccination programs) among other duties. During public health emergencies, the provision of regular public health services can be interrupted [14–16]. Depending on the type and scope of the emergency and the size and capability of the public health workforce, response may represent a burden that negatively affects the efficacy and efficiency of regular functions or professional roles. The purpose of this study was to quantify the impacts of the COVID-19 response on the public health workforce’s program areas, job functions, and work hours.

## Materials and methods

We conducted a cross-sectional survey of individuals working in public health during the ongoing COVID-19 pandemic response. The survey assessed the public health workforce across a variety of domains such as professional experience (i.e., training, years of experience, content expertise, job functions), mental and physical health status (i.e., generalized anxiety, depression, burnout), and career plans (i.e., pre-pandemic vs. current career plans) [17] (See Supporting Information). We pilot tested the survey for clarity and content with a group of epidemiologists working in a large, local public health department and revised the survey based on this feedback. The final survey was distributed using Qualtrics through professional networks and professional association listservs (i.e., the American Public Health Association’s Epidemiology Section) to potential respondents (n = 3,000) with either an academic degree in a field related to public health or a professional role in an academic or governmental public health department. Results presented here include responses collected from August 23 –October 5, 2020.

Data were analyzed using SAS v.9.4 (SAS Institute; Cary, NC). Descriptive statistics including frequency and percentages, percent decrease/increase, and means were calculated. We compared reported program areas, job functions, and hours worked in the pre-pandemic period (January 2020) vs. the mid-pandemic period (August–October 2020) using McNemar’s test. Responses to three qualitative questions (best practices, routine services not able to be done, and suggestions for improvement to the assessment itself) were downloaded into a Microsoft Excel spreadsheet (Redmond, WA) and inductively coded by a trained graduate student to identify themes. This survey and all related materials were reviewed by the University of Delaware Institutional Review Board. Informed consent was not required as the research was determined to be exempt under 45 CFR46.101(b) of the U.S. Department of Health and Human Services regulations for human subjects research (IRB# 1641836–1).

## Results

### Sociodemographic and professional experience

From August 23 through October 5, 2020, 298 individuals from 31 U.S. States and the District of Columbia responded to our survey. Respondents were mostly female (82%), white, non-Hispanic (74%), between the ages of 18 and 39 years (60%) and identified as public health practitioners (84%). Academics (10%) and epidemiologists in other fields (e.g., non-profit, private industry, clinical; 4%) rounded out the sample (2% were missing). Of these, 16 (5%) were new public health hires since January 2020. Over half of the sample had between 1 and 9 years of experience in public health (51%), and over one-third had 10 or more years of experience (38%).

### Public health content areas

**Fig 1:**
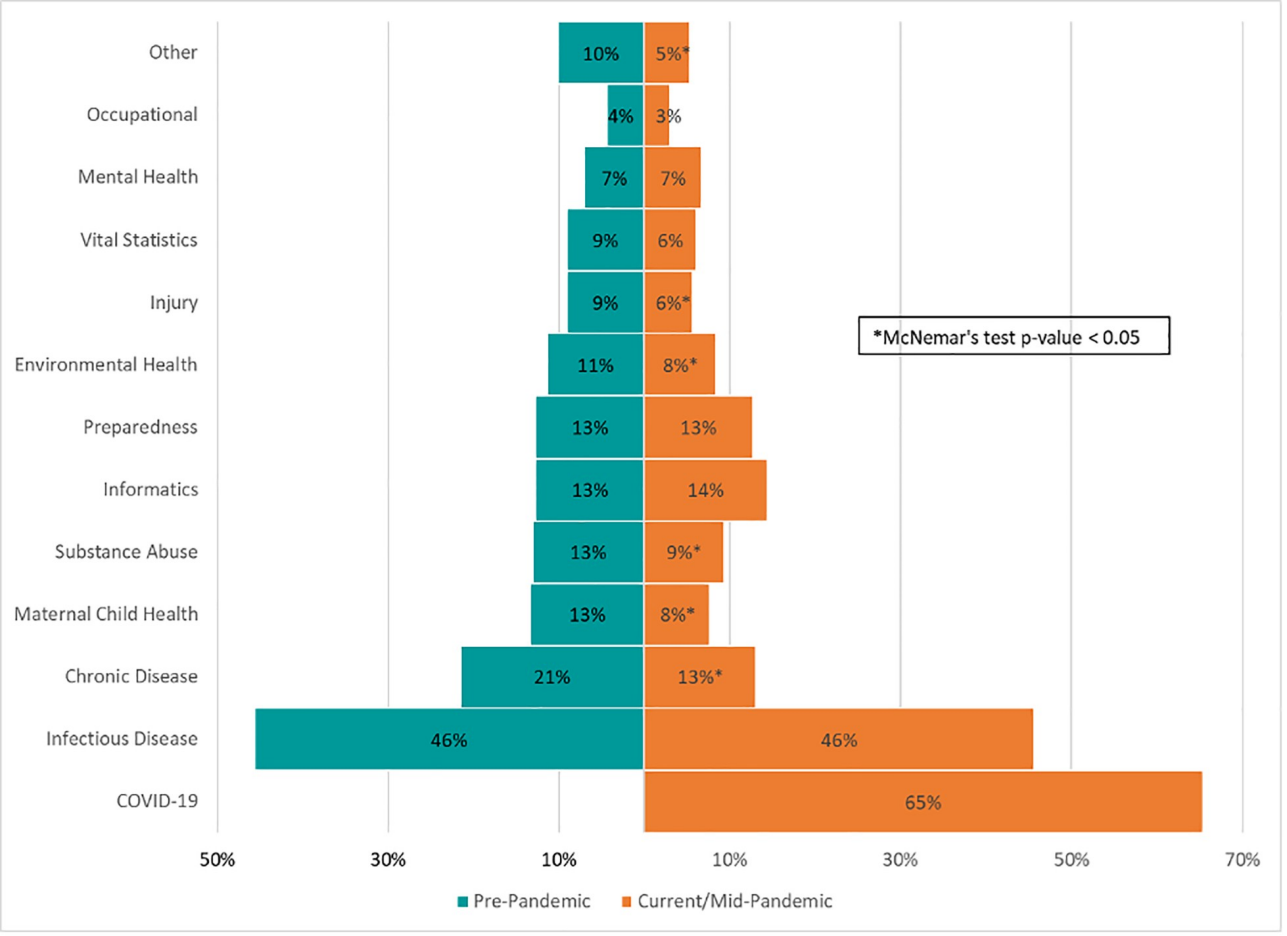
Types of public health content areas pre-pandemic vs. mid-pandemic–U.S., August 23–October 5, 2020.

Many essential public health content expertise areas have had staffing redirected to respond to the COVID-19 pandemic (Fig 1). When asked to compare pre-pandemic content expertise (January 2020) to mid-pandemic content expertise (August—October 2020), respondents reported reductions in chronic disease (percent decrease: 39%), maternal-child health (percent decrease: 42%), substance abuse (percent decrease: 28%), environmental health (percent decrease: 26%), injury (percent decrease: 37%), and other program areas, including HIV/sexually transmitted infections/tuberculosis/sexual health programs, social epidemiology/health disparities programs, among others (percent decrease: 47%).

### Public health content expertise and programmatic functions

Among the 282 (95%) respondents who worked in public health in January 2020 (i.e., not new hires), 60% reported having no changes in the number of content expertise areas or programmatic functions that they were responsible for pre-pandemic vs. mid-pandemic; however, 26% reported an increase in both during this period. For the 60% who did not have an increase or decrease in the number of responsibilities, the area of expertise or the program function where they worked often changed. For example, a respondent’s role changed from a single pre-pandemic content expertise of 100% environmental health to a single mid-pandemic content expertise of 100% COVID-19).

As previously shown, among all 298 respondents, several content expertise areas showed a decrease in staffing since the start of the pandemic. Notably, the total number of respondents working in infectious disease and preparedness remained constant. No content areas except COVID-19 showed increases in staffing (Fig 1). The total number of program areas covered increased from 509 pre-pandemic to 607 mid-pandemic or 1.7 to 2.0 program areas per person.

**Fig 2:**
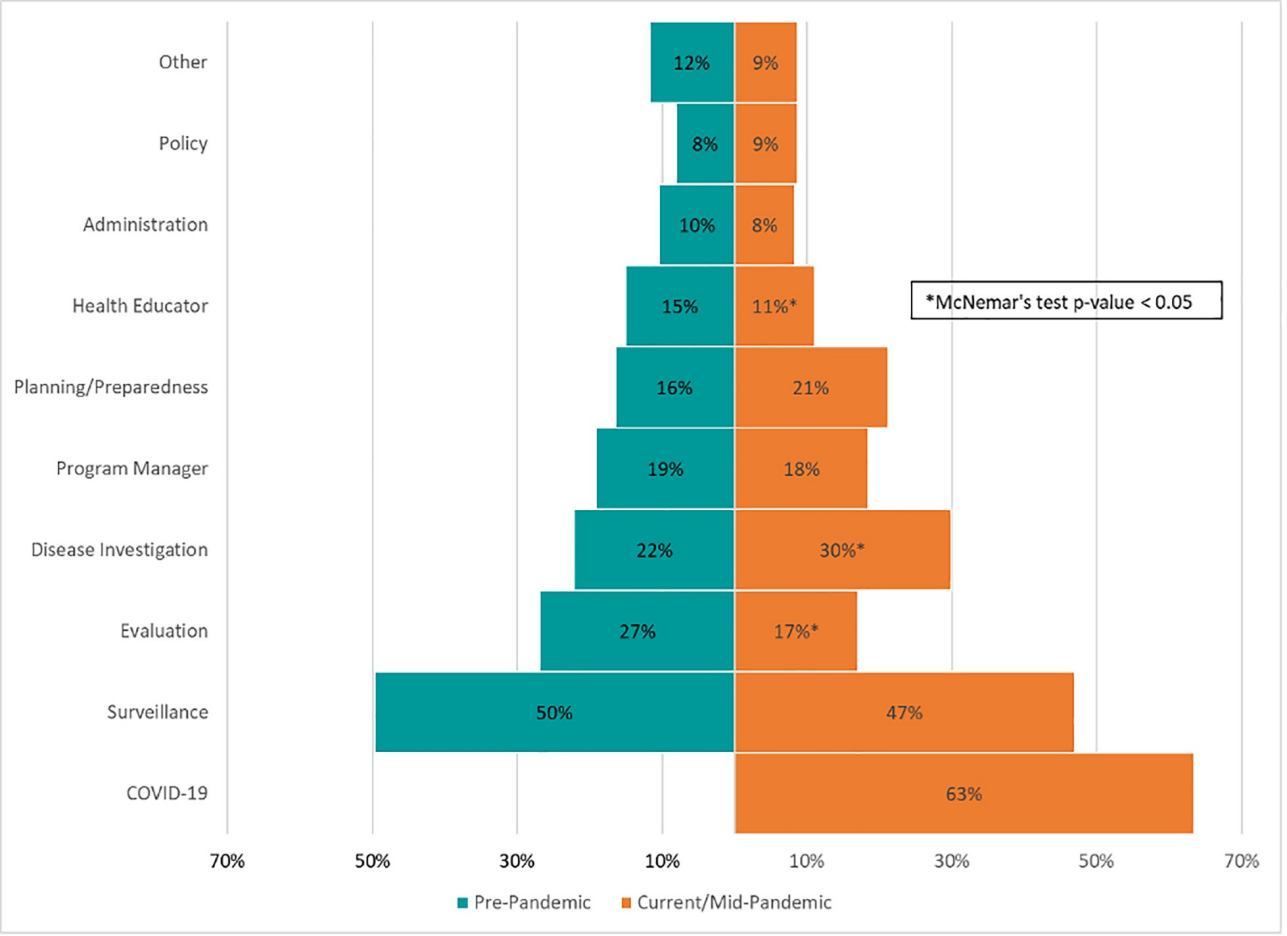
Types of public health functions covered pre-pandemic vs. mid-pandemic–U.S., August 23–October 5, 2020.

The total number of respondents filling surveillance, program manager, planning/preparedness, administration, and policy roles did not change pre-pandemic to mid-pandemic. The programmatic functions that saw a significant decline were program evaluation (percent decrease: 36%) and health education (27%). Disease investigation significantly increased (percent increase: 35%; Fig 2). The total number of programmatic functions/roles increased from 536 pre-pandemic to 697 mid-pandemic or 1.8 to 2.3 per person.

Table 1 shows the pre-pandemic content expertise areas and programmatic functions of workers that were reassigned or given additional responsibilities as part of the COVID-19 response. For content expertise, the majority of the mid-pandemic COVID-19 workers (47%) were infectious disease practitioners prior to the COVID-19 pandemic. Other reassignments to the COVID-19 workforce included those previously working in chronic disease (23%), substance abuse (16%), and maternal-child health (15%). For programmatic functions, over half of the mid-pandemic COVID-19 workers (55%) came from surveillance. Other programmatic functions with workers reassigned to the COVID-19 response included evaluation (29%), disease investigation (27%), and planning/preparedness (21%).

**Table 1:** Pre-pandemic expertise of individuals working on COVID-19 mid-pandemic–U.S., August 23–October 5, 2020.

**Content Expertise**	**N**	**% of 195**
Infectious Disease	92	47.18%
Chronic Disease	44	22.56%
Substance Abuse	31	15.90%
Maternal-Child Health	29	14.87%
Environmental Health	28	14.36%
Informatics	26	13.33%
Preparedness	25	12.82%
Injury	21	10.77%
Vital Statistics	21	10.77%
Mental Health	17	8.72%
Occupational	10	5.13%
Other	17	8.72%
None	22	11.28%
**Programmatic Functions**	**N**	**% of 189**
Surveillance	103	54.50%
Evaluation	55	29.10%
Disease Investigation	51	26.98%
Planning/Preparedness	39	20.63%
Program Manager	35	18.52%
Health Educator	31	16.40%
Administration	20	10.58%
Policy	19	10.05%
Other	20	10.58%
None	15	7.94%

Table 2 shows content areas and programmatic functions unchanged from pre- to mid-pandemic, that is, individuals working in the same content expertise areas and programmatic functions at both time points. Over half of individuals working in infectious disease (69 of 136), substance abuse (20 of 39), and preparedness 20 of 38), and almost two-thirds of informatics (23 of 38) and vital statistics (17 of 27) were reassigned to work in other content areas during the pandemic. Areas that saw the greatest proportion of workers reassigned to COVID-19 response included occupational health (11 of 13), chronic disease (51 of 64), and injury (19 of 27). Individuals working in administration (27 of 31), evaluation (66 of 80), disease investigation (54 of 66), and planning/preparedness programmatic (38 of 49) roles were also likely to be reassigned to the COVID-19 response, demonstrating how key public health areas are losing their content experts to the pandemic response.

**Table 2:** Number maintaining content and programmatic expertise during pandemic–U.S., August 23–October 5, 2020.

**Content Expertise**	**N** *	**Pre-COVID N**	**% Pre-Pandemic Workers Retained**
Infectious Disease	67	136	49.26%
Substance Abuse	19	39	48.72%
Preparedness	18	38	47.37%
Informatics	15	38	39.47%
Vital Statistics	10	27	37.04%
Other	11	30	36.67%
MCH	13	40	32.50%
Environmental Health	11	34	32.35%
Injury	8	27	29.63%
Chronic	13	64	20.31%
Occupational Health	2	13	15.38%
**Programmatic Function**	**N** *	**Pre-COVID N**	**% Pre-Pandemic Workers Retained**
Surveillance	105	148	70.95%
Other	18	35	51.43%
Program Manager	24	57	42.11%
Policy	6	24	25.00%
Health Educator	11	45	24.44%
Planning/Preparedness	11	49	22.45%
Disease Investigation	12	66	18.18%
Evaluation	14	80	17.50%
Administration	4	31	12.90%

*pre-COVID area/role = post-COVID area/role.

### Work hours

**Fig 3:**
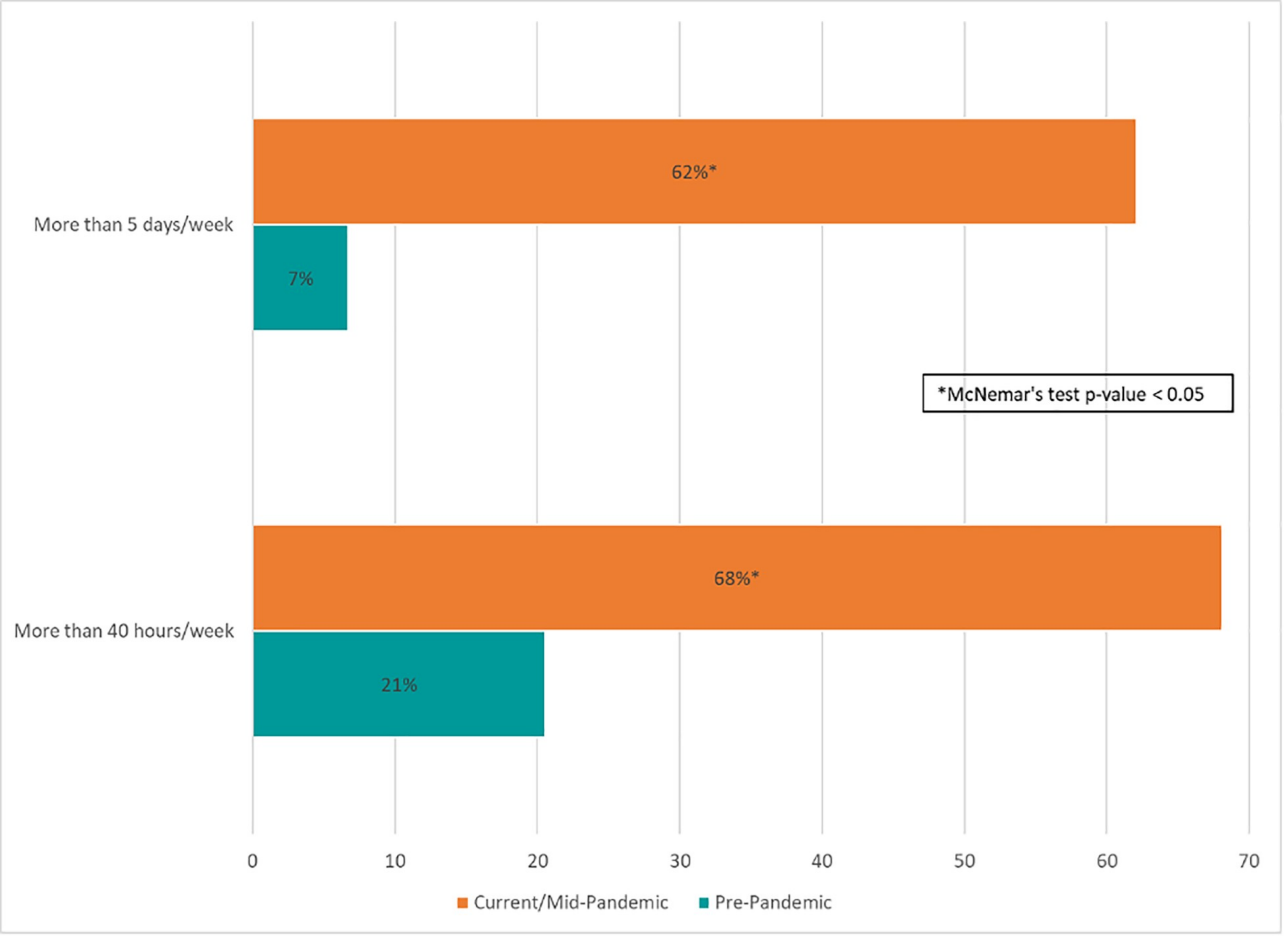
Days and hours worked per week pre-pandemic vs. mid-pandemic–U.S., August 23–October 5, 2020.

Fig 3 shows the average number of working hours and days per week reported by survey respondents pre-pandemic vs. mid-pandemic. Among the 282 individuals working in public health in January 2020, there was a significant increase in those reporting working overtime since the start of the pandemic. Mid-pandemic, about two-thirds said they were working more than 40 hours and more than five days per week, compared to 21% and 7%, respectively, pre-pandemic. Average days worked per week increased by 0.8 days and average hours worked per week increased by 11.2, compared to pre-pandemic.

## Discussion

The large-scale, long-term public health emergency response to the COVID-19 pandemic has placed an unsustainable burden on the U.S. public health workforce, which began the pandemic response severely underfunded and understaffed. Prior research has demonstrated direct linkages between per capita funding for public health and public health workforce development, and public health departments’ abilities to provide essential public health services [13, 18–20]. Since 2008, the public health workforce has shrunk by 20%, with 62% of local health departments seeing their budgets flat line, or reduce over time [21–23]. U.S. public health systems cannot currently maintain many essential public health services while responding to the COVID-19 pandemic.

According to qualitative responses, many routine duties and services were no longer able to be done due to the burden of COVID-19 response, including investigations related to other communicable diseases, foodborne outbreaks, public health surveillance and evaluation, and non-communicable disease response. The most frequently mentioned routine duties that were interrupted included work on other communicable diseases besides COVID-19, including sexually transmitted infections, enteric diseases, and Hepatitis B and C. Foodborne outbreaks were specifically mentioned by respondents, who pointed out that there was little capacity to conduct surveillance, outbreak investigations, or inspections. Routine disease surveillance and evaluations of surveillance programs were also reported to have been interrupted due to COVID-19 response, even for critical functions such as perinatal diseases and maternal-child health outcomes. Work related to blood lead investigations, vector-borne diseases, and immunizations were also interrupted. Little time was available for chronic diseases, which may also be due in part to closures or limitations in the use of public health facilities, which means that walk-in programs for addiction, in-person meetings with stakeholder coalitions, and regular maternal-child health programs could no longer be provided. Grant-funded work related to disease prevention, including opioid abuse prevention, as well as the investigation of non-fatal overdoses, stopped in some jurisdictions due to the COVID-19 response.

In addition to funding and workforce shortages among public health staff, public health leaders have faced widespread pressure from outside forces, which have led to a reduction in the public’s trust in public health experts [24] and a number of firings, resignations, and retirements [25]. By December 2020, 20 states had lost their state-level public health director [25], and 37 city and county health officials had left office [26]. This left many communities without public health leadership just as they embarked on an unprecedented vaccination program amid a COVID-19 surge.

While this study and prior research addresses the impacts of inadequate funding and workforce challenged on the provision of essential public health services in the U.S., the World Health Organization identified challenges to maintaining essential health services globally in March 2020 guidance [27]. Disruptions to childhood vaccination programs were singled out, with 85% of 61 responding countries reporting disruptions to immunization programs related to COVID-19 due to shortages in personal protective equipment, low availability of healthcare workers, and travel restrictions [28]. According to the Centers for Disease Control and Prevention [29], 41 countries were planning to cancel or delay measles vaccination campaigns for 2020 and 2021.

This cross-sectional study has several important limitations. First, our results are not representative of the U.S. public health workforce. Female, non-Hispanic, White, and respondents under 40 years of age were overrepresented in this survey compared to the most recent estimates of the public health workforce [30]. Second, our sample was non-probabilistic. Although respondents were from 25 U.S. States and the District of Columbia and worked in a wide range of health department functions (e.g., infectious disease, substance abuse, public health preparedness, informatics, maternal and child health, environmental health, injury, chronic disease, vital statistics, and occupational health), health departments in the U.S. vary widely in terms of their staffing, programs, and governance, which limits the generalizability of any findings. Response bias is also possible if public health staff who had changed roles or functions due to COVID-19 were more likely to respond than those who did not change roles or functions. Recall bias is possible when asking respondents to retrospectively report job functions and work hours from January 2020. Results presented include responses through October 5, 2020, approximately six months into the pandemic response. Therefore, responses may not represent the current state of the public health workforce, as cases of COVID-19 continued to rise through early 2021.

The provision of essential public health services has often been anecdotally described as invisible when working well [31]. However, as the COVID-19 pandemic is clearly demonstrating, current policies related to funding and professional development of the U.S.’s public health workforce are inadequate for supporting an effective response to a public health emergency while maintaining the provision of essential population health services. What remains unknown, but critically important to quantify, are the impacts to the public’s health that will result from these interruptions during the COVID-19 pandemic response. Going forward, no matter the extent or nature of the public health emergency, the public health system must be robust enough to continue the simultaneous provision of essential public health services.

## Conclusion

The provision of essential public health functions and services has been interrupted by the public health response to COVID-19. Public health staff are taking on more responsibilities and covering more program areas; with the response to COVID-19 shifting staff from evaluation and health education to disease investigation. Qualitative investigations of future impacts, with a focus on potential inequities among workforce subgroups, are needed. The burden of the COVID-19 response on those working in public health practice is likely to impact the public health workforce, and by extension, public health, for years to come.

## Supporting information

S1 Text Survey questions. (DOCX)

S1 Dataset Survey responses. (XLSX)
