# Supplementary material for: The impact of the COVID-19 response on the provision of other public health services in the U.S.: A cross sectional study
Source: PLoS One. 2021 Oct 14;16(10):e0255844. doi: 10.1371/journal.pone.0255844 (PMC8516286; doi:10.1371/journal.pone.0255844)
Supplement: Supplementary file 1 [file pone.0255844.s001.docx]

Epidemiology Workforce COVID Impact Assessment

Start of Block: Consent

Q1 
The public health response to the SARS-CoV-2 (COVID-19) pandemic is unprecedented in recent history; as such, it is taking a toll on the health and well-being of essential workers. As the response will likely continue for months and intensify in some areas, understanding the on-going impacts on public health practitioners will allow us to modify and adapt policies and procedures to allow for a more sustained and healthy workforce. 

 

The goal of this survey is to systematically assess the impacts of the COVID-19 response on the mental and physical health of epidemiologists and other public health workers; identify risk and protective factors for burnout within workplaces and communities; and understand short- and long-term career decisions that may result from the pandemic and response. 

 

The target population of this assessment includes epidemiologists and those working in public health doing epidemiology-related functions who have been directly and indirectly impacted by the public health response to COVID-19. This survey was reviewed by the University of Delaware Institutional Review Board and determined to be exempt (IRB #1641836-1).

 

Do you wish to participate in this assessment?

 

- Yes  (1) 

- No  (2) 

Skip To: End of Survey If Q1 = 2

End of Block: Consent

Start of Block: Background Information

Q2 What is your age?

- 18-29  (1) 

- 30-39  (2) 

- 40-49  (3) 

- 50-64  (4) 

- 65-74  (5) 

- 75-84  (6) 

- 85+  (7) 

- Prefer not to say  (8) 

Q3 What is your gender?

- Male  (1) 

- Female  (2) 

- Other (please specify)  (3) ________________________________________________

- Prefer not to say  (4) 

Q4 Are you of Spanish or Latino origin?

- Yes  (1) 

- No  (2) 

- Prefer not to say  (3) 

Q5 What would best describe your race?

- American Indian/Alaskan Native  (1) 

- Asian  (2) 

- Black  (3) 

- Native Hawaiian/Other Pacific Islander  (4) 

- White  (5) 

- Other (please specify):  (6) ________________________________________________

- Prefer not to say  (7) 

Q6 What is your marital status?

- Now married  (1) 

- Widowed  (2) 

- Divorced  (3) 

- Seperated  (4) 

- Never Married  (5) 

- Other (please specify):  (6) ________________________________________________

- Prefer not to say  (7) 

Q7 How many people currently reside in your household including yourself?

- Adults (18 years and older; including yourself):  (1) ________________________________________________

- Children (less than 18 years):  (2) ________________________________________________

- Prefer not to say  (3) 

Q8 What is your state and county of residence?

State (1) 

County (2) 

▼ Alabama (1) ... Wyoming ~ Weston County (3192)

End of Block: Background Information

Start of Block: Public Health Role and Training

Q9 Position and Agency

- Title/Position  (1) ________________________________________________

- Agency or Organization  (2) ________________________________________________

- Program or Department  (3) ________________________________________________

- Full Time or Part Time (enter FT or PT)  (4) ________________________________________________

Q10 What type of field do you currently work in?

- Public Health Practice  (1) 

- Academic  (2) 

- Clinical Setting  (3) 

- Other (please specify):  (4) ________________________________________________

Q11 
How many years of experience do you have in public health practice?

- Less than 1 year  (1) 

- 1-4 years  (2) 

- 5-9 years  (3) 

- 10-14 years  (4) 

- 15+ years  (5) 

Q12 In which of the following **epidemiology program areas** do you have experience**?** (Select all that apply)

- Chronic Disease	  (1) 

- Environmental Health  (2) 

- Genomics  (3) 

- Infectious Disease   (4) 

- Informatics  (5) 

- Injury  (6) 

- Maternal and Child Health  (7) 

- Mental Health  (8) 

- Occupational Health  (9) 

- Preparedness  (10) 

- Substance Abuse  (11) 

- Vital Statistics  (12) 

- Other (please specify):   (13) ________________________________________________

- None, I have not worked in epidemiology  (14) 

Q13 In which of the following **public health practice roles** do you have experience? (Select all that apply)

- Administration  (1) 

- Benefits coordinator  (2) 

- Disease investigator  (3) 

- Evaluation  (4) 

- Health educator  (5) 

- Planning/preparedness  (6) 

- Policy  (7) 

- Program manager  (8) 

- Surveillance  (9) 

- Other (please specify):  (10) ________________________________________________

- None, I have not worked in public health practice  (12) 

Q14 What was your pre-pandemic **epidemiology program area**? (Select all that apply)

- Chronic Disease	  (1) 

- Environmental Health  (2) 

- Genomics  (3) 

- Infectious Disease   (4) 

- Informatics  (5) 

- Injury  (6) 

- Maternal and Child Health  (7) 

- Mental Health  (8) 

- Occupational Health  (9) 

- Preparedness  (10) 

- Substance Abuse  (11) 

- Vital Statistics  (12) 

- Other (please specify):   (13) ________________________________________________

- None, I did not work in an epidemiology program area  (14) 

Q15 What was your pre-pandemic **public health practice role**? (Select all that apply)

- Administration  (1) 

- Benefits coordinator  (2) 

- Disease investigator  (3) 

- Evaluation  (4) 

- Health educator  (5) 

- Planning/preparedness  (6) 

- Policy  (7) 

- Program manager  (8) 

- Surveillance  (9) 

- Other (please specify):  (10) ________________________________________________

- None, I did not work in public health practice  (11) 

Q16 What is your current/mid-pandemic **epidemiology program area**? (Select all that apply)

- Chronic Disease	  (1) 

- COVID-19 Specific  (14) 

- Environmental Health  (2) 

- Genomics  (3) 

- Infectious Disease   (4) 

- Informatics  (5) 

- Injury  (6) 

- Maternal and Child Health  (7) 

- Mental Health  (8) 

- Occupational Health  (9) 

- Preparedness  (10) 

- Substance Abuse  (11) 

- Vital Statistics  (12) 

- Other (please specify):   (13) 

- None, I do not work in an epidemiology program area  (15) 

Q17 What is your current/mid-pandemic **public health practice role**? (Select all that apply)

- Administration  (1) 

- Benefits coordinator  (2) 

- COVID-19 Specific  (11) 

- Disease investigator  (3) 

- Evaluation  (4) 

- Health educator  (5) 

- Planning/preparedness  (6) 

- Policy  (7) 

- Program manager  (8) 

- Surveillance  (9) 

- Other (please specify):  (10) ________________________________________________

- None, I do not work in a public health practice  (12) 

Q18 
What is the highest degree or level of education you have completed?

- Less than high school diploma  (1) 

- High school degree or equivalent  (2) 

- Technical certificate/training (please specify):  (3) ________________________________________________

- Associates degree (e.g., AA, AS)  (4) 

- Bachelor’s degree (e.g., BA, BS, BSN), Please specify subject:  (5) ________________________________________________

- Master’s degree (e.g., MPH, MSPH, MS, MBA, NP, MSN), Please specify degree and subject:  (6) ________________________________________________

- Professional degree beyond a bachelor’s degree (e.g., MD, DDS, DVM, DNP), please specify degree and specialty if applicable:  (7) ________________________________________________

- Doctorate degree (e.g., PhD, DrPH), please specify degree and subject:  (8) ________________________________________________

Q20 Do you have any other certifications or training related to your role in public health practice?

- CHES  (1) 

- CPH  (2) 

- GISP  (3) 

- CIC  (4) 

- Other (please specify):  (5) ________________________________________________

Q19 In January 2020, which best describes your career plans or trajectory with regards to public health practice?

- Leave or retire **during 2020**  (1) 

- Remain **during 2020**, but leave or retire in a **year or two**  (6) 

- Remain for **3 or more years**  (2) 

- Undecided  (4) 

- Not Applicable  (10) 

- Prefer not to say  (5) 

Q21 As of today, what are your career plans or trajectory with regards to public health practice?

- Leave or retire **during 2020**  (1) 

- Remain **during 2020**, but leave or retire in a **year or two**  (6) 

- Remain for **3 or more years**  (2) 

- Undecided  (4) 

- Not Applicable  (10) 

- Prefer not to say  (5) 

End of Block: Public Health Role and Training

Start of Block: Personal Health and Well-being

Q22 Thinking about your physical health, which includes physical illness and injury, for how many days during the past 30 days was your physical health not good? 

- Number of days:  (1) ________________________________________________

- None   (2) 

- Don't know/ not sure  (3) 

- Prefer not to say  (4) 

Q23 Thinking about your mental health, which includes stress, depression, and problems with emotions, for how many days during the past 30 days was your mental health not good?

- Number of days:  (1) ________________________________________________

- None   (2) 

- Don't know/ not sure  (3) 

- Prefer not to say  (4) 

Q24 During the past 30 days, for about how many days did poor physical or mental health keep you from doing your usual activities, such as self-care, work, or recreation?

- Number of days:  (1) ________________________________________________

- None   (2) 

- Don't know/ not sure  (3) 

- Prefer not to say  (4) 

Q25 Over the last 2 weeks, how often have you been bothered by the following problems? 

|  | Not at all (1) | Several days (2) | Over half the days (3) | Nearly every day (4) |
| --- | --- | --- | --- | --- |
| Feeling nervous, anxious, or on edge  (1) |  |  |  |  |
| Not being able to stop or control worrying (2) |  |  |  |  |
| Worrying too much about different things (3) |  |  |  |  |
| Trouble relaxing (4) |  |  |  |  |
| Being so restless that it's hard to sit still (5) |  |  |  |  |
| Becoming easily annoyed or irritable (6) |  |  |  |  |
| Feeling afraid as if something awful might happen   (7) |  |  |  |  |
| Little interest or pleasure in doing things (8) |  |  |  |  |
| Feeling down, depressed, or hopeless (9) |  |  |  |  |

Q26 In your opinion, what was the **most difficult** two-week period of 2020? Please select the **start date** of that two-week period: 
 
 
 

Qualtrics.SurveyEngine.addOnload(function ()
{
  var qid = this.questionId;
  var calid = qid + '_cal';
  var y = QBuilder('div');
  $(y).setStyle({clear:'both'});
  var d = QBuilder('div',{className:'yui-skin-sam'},[
    QBuilder('div', {id:calid}),
    y
  ]);
  
  var c = this.questionContainer;
  c = $(c).down('.QuestionText');
  c.appendChild(d);
  var cal1 = new YAHOO.widget.Calendar(calid); 
  cal1.render(); 
  var input = $('QR~' + qid);
  $(input).setStyle({marginTop: '20px',width: '150px'});
  var p =$(input).up();
  var x = QBuilder('div');
  $(x).setStyle({clear:'both'});
  p.insert(x,{position:'before'});
    cal1.selectEvent.subscribe(function(e,dates){
    var date = dates[0][0];
    if (date[1] < 10)
        date[1] = '0' + date[1];
    if (date[2] < 10)
        date[2] = '0' + date[2];
    input.value = date[1] +'-'+date[2]+'-'+date[0];
  })
});

________________________________________________________________

Q27 Over the two-week period you indicated as **being most difficult in 2020**, how often have you been bothered by the following problems? 

|  | Not at all (1) | Several days (2) | Over half the days (3) | Nearly every day (4) |
| --- | --- | --- | --- | --- |
| Feeling nervous, anxious, or on edge  (1) |  |  |  |  |
| Not being able to stop or control worrying (2) |  |  |  |  |
| Worrying too much about different things (3) |  |  |  |  |
| Trouble relaxing (4) |  |  |  |  |
| Being so restless that it's hard to sit still (5) |  |  |  |  |
| Becoming easily annoyed or irritable (6) |  |  |  |  |
| Feeling afraid as if something awful might happen   (7) |  |  |  |  |
| Little interest or pleasure in doing things (8) |  |  |  |  |
| Feeling down, depressed, or hopeless (9) |  |  |  |  |

Q28 Overall, based on your definition of burnout, how would you rate your level of burnout today?

- I enjoy my work. I have no symptoms of burnout.  (1) 

- Occasionally I am under stress, and I don’t always have as much energy as I once did, but I don’t feel burned out.   (2) 

- I am definitely burning out and have one or more symptoms of burnout, such as physical and emotional exhaustion.   (3) 

- The symptoms of burnout that I’m experiencing won’t go away. I think about frustration at work a lot.  (4) 

- I feel completely burned out and often wonder if I can go on. I am at the point where I may need some changes or may need to seek some sort of help.  (5) 

End of Block: Personal Health and Well-being

Start of Block: Work-life Balance Functioning

Q29 Considering your work and personal life: **In the past**, when you encounter difficulties, what is the source that you ever received **comfort** and **caring**? (Select all that apply)

- No source  (1) 

- Spouse/Partner(s)  (2) 

- Other family members  (3) 

- Friends  (4) 

- Relatives  (5) 

- Colleagues  (6) 

- Companies  (7) 

- Official or semi-official organizations, such as, parties, leagues and trade union  (8) 

- Unofficial organizations, such as, religion, social group and etc.  (9) 

- Others (please specify)  (10) ________________________________________________

Q30 Considering your work and personal life: In the past, when you encounter difficulties, what is the source that you ever received either **economic support** or **practical problem-solving** help? (Select all that apply)

- No source  (1) 

- Spouse/Partner(s)  (2) 

- Other family members  (3) 

- Friends  (4) 

- Relatives  (5) 

- Colleagues  (6) 

- Companies  (7) 

- Official or semi-official organizations, such as, parties, leagues and trade union  (8) 

- Unofficial organizations, such as, religion, social group and etc.  (9) 

- Others (please specify)  (10) ________________________________________________

Q31 For households with children, what type of child care arrangement does your family use currently? (Select all that apply)

- Cared for in our home by parents/family/sitter  (1) 

- Informal arrangement with neighbors/family  (2) 

- Child care center/ preschool  (3) 

- Home-based care  (4) 

- School (K-12)  (8) 

- Other (please specify)  (5) ________________________________________________

- Children are of age that do not require childcare  (6) 

- Prefer not to say  (7) 

Q32 Are you or anyone in your household caring for a sick family member or providing elder care?

- Yes  (1) 

- No  (2) 

- Unsure  (3) 

- Prefer not to say  (4) 

Q33 Do you or anyone in your household have a medical condition that increases risk for severe illness from COVID-19 [i.e., cancer, chronic kidney disease, COPD, immunocompromised state (weakened immune system), obesity (body mass index [BMI] of 30 or higher), serious heart conditions, sickle cell disease, type 2 diabetes mellitus]?

- Yes  (1) 

- No  (2) 

- Unsure  (3) 

- Prefer not to say  (4) 

Q34 What do you feel is the level of your financial stress today?

- Overwhelming stress  (1) 

- Above average stress  (2) 

- Average stress  (3) 

- Some stress  (4) 

- No stress at all  (5) 

Q35 How often do you?

|  | Frequently (1) | Occasionally (2) | Rarely (3) | Never (4) | It never occurred to me (5) |
| --- | --- | --- | --- | --- | --- |
| Eat regularly (e.g. breakfast, lunch and dinner) (1) |  |  |  |  |  |
| Eat healthy foods (2) |  |  |  |  |  |
| Exercise consistently (3) |  |  |  |  |  |
| Get regular medical care for prevention (4) |  |  |  |  |  |
| Get medical care when necessary (5) |  |  |  |  |  |
| Take time off when sick (6) |  |  |  |  |  |
| Feel you can take time off from work (7) |  |  |  |  |  |
| Get enough sleep (8) |  |  |  |  |  |

Q36 In a week, how many hours did you work **pre-pandemic** vs. **the past week**?

|  | 0 | 20 | 40 | 60 | 80 | 100 |
| --- | --- | --- | --- | --- | --- | --- |

| **Pre-Pandemic** number of hours () |  |
| --- | --- |
| In the **past week** number of hours () |  |

Q37 How many days a week did/do you typically work **pre-pandemic** and **now**?

|  | 0 | 1 | 1 | 2 | 2 | 3 | 3 | 4 | 4 | 5 | 5 | 6 | 6 | 7 |
| --- | --- | --- | --- | --- | --- | --- | --- | --- | --- | --- | --- | --- | --- | --- |

| Days per week **pre-pandemic** () |  |
| --- | --- |
| Days per week **now** () |  |

Q38 Have you or anyone within your agency received any personal threats regarding COVID-19 from the public or anyone outside of your agency?

- Yes  (1) 

- No  (2) 

- Unsure  (3) 

- Prefer not to say  (4) 

Q39 During the COVID-19 response, which of the following has your workplace adopted as safety measures? 

(Select all that apply)

- Required masks  (1) 

- Required face shields/protection  (2) 

- Social distancing indoors  (3) 

- Social distancing outdoors  (4) 

- Adapted food safety practices  (5) 

- Telework options (working remotely)  (6) 

- Flexible work schedules / rotations  (7) 

- Additional cleaning procedures  (8) 

- Other, (please describe):  (9) ________________________________________________

Q40 Are there any best practices observed in the workplace that you’d like to highlight (e.g. healthy food options in the Emergency Operations Center, an online chat communication platform like skype/google for communication)? *Please be as specific as possible.*

 

________________________________________________________________

________________________________________________________________

________________________________________________________________

________________________________________________________________

________________________________________________________________

Q41 During the response to COVID-19, what routine duties or services have/are not able to be done (i.e., what pre-pandemic public health services are not getting done now, like foodborne investigations)? *Please be as specific as you feel comfortable.*

 

________________________________________________________________

________________________________________________________________

________________________________________________________________

________________________________________________________________

________________________________________________________________

Q42 Do you have any specific suggestions or feedback about this assessment regarding the questions that were asked or any that were absent? *Please be as specific as possible.*

 

________________________________________________________________

________________________________________________________________

________________________________________________________________

________________________________________________________________

________________________________________________________________

Q43 Would you be willing to participate in a phone or virtual interview as a key informant?

- Yes  (1) 

- No  (2) 

Q44 Would you allow this research team to contact you in the future as part of a longitudinal study?

- Yes  (1) 

- No  (2) 

Display This Question:

If Q43 = 1

Or Q44 = 1

Q45 Since you indicated that you are willing to **participate in a phone or virtual interview** or **be a part of a longitudinal study** or **both**, please provide your name and preferred email address. Thank you!

- Name  (4) ________________________________________________

- Preferred Email  (5) ________________________________________________

End of Block: Work-life Balance Functioning
